# Fossil Bacteria

**Published:** 1897-12

**Authors:** 


### Fossil Bacteria.

M. B. Renault (*Ann. des Sciences Natur.*; ref *Brit. Pharm. Jour.*) has long worked at the indications of bacteria found in geological strata, and now publishes the general results of his observations in a paper illustrated with a large number of drawings. As might be expected from their simple structure, bacteria appear to have been coeval with the first appearance of organic life on earth, the coccoid form being apparently earlier than the bacillar. Indications of their presence are found in bone, teeth, scales and coprolites, as well as abundantly in vegetable tissues, the spores and sporanges of ferns appearing to have been especially subject to their attacks. The species are, as a rule, distinct from those at present in existence.—*B. in American Medico-Surgical Bulletin.*

